# Supplementary material for: Identification of photoperiod-induced specific miRNAs in the adrenal glands of Sunite sheep (Ovis aries)
Source: Front Vet Sci. 2022 Jul 22;9:888207. doi: 10.3389/fvets.2022.888207 (PMC9354845; doi:10.3389/fvets.2022.888207)
Supplement: Supplementary file 1 [file Data_Sheet_1.ZIP › Supplementary file/miRDeep2 file.zip!/miRDeep2 file/Novel_Structure/SPLP42A1/Novel_582.pdf]

Provisional ID	:	NC_019464.2_13076
Score total	:	1.6
Score for star read(s)	:	-1.3
Score for read counts	:	0
Score for mfe	:	1.3
Score for randfold	:	1.6
Score for cons. seed	:	
Total read count	:	128
Mature read count	:	128
Loop read count	:	0
Star read count	:	0

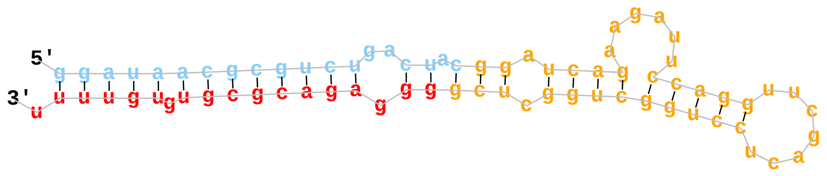

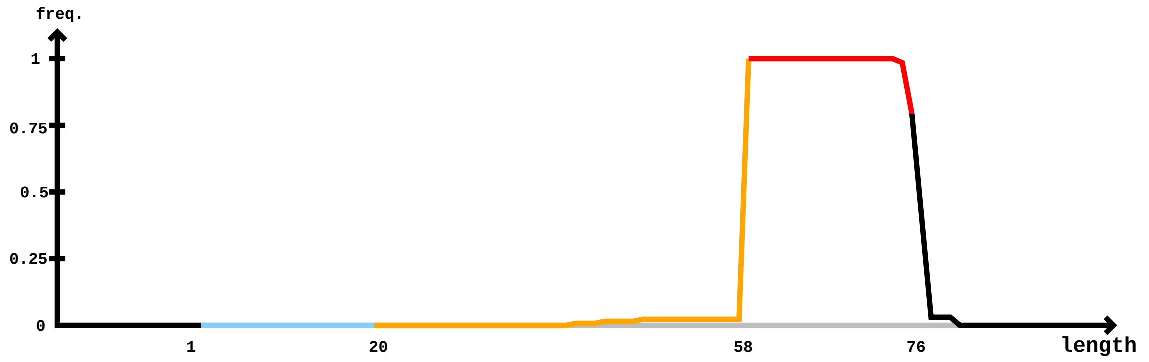


```
                     Star                                                           Mature
5'- ccaguggcgcaauggauaacgcgucugacuacggaucagaagauuccagguucgacuccuggcuggcucggggagacgcguguguuuuuguugcaucccaugcaauac   -3'    exp
    ...((((.((((((((..((((((((..((.(((.((((......(((((.......))))))))).))))).))))))))).....))))))))...)))).......    reads       mm          sample
    ....................................................cgacuccuggcuggcucggggagacgcgugugguuu.....................    1           0           seq
    .......................................................cuccuggcuggcucggggagacgcgugugu.......................    1           0           seq
    ...........................................................uggcuggcucggggagacgcgugugu.......................    1           0           seq
    ......................................................................gggagacgcguguguuu.....................    24          0           seq
    ......................................................................gggagacgcguguguuuu....................    89          0           seq
    ......................................................................gggagacgcguguguuuuu...................    11          0           seq
    ......................................................................gggagacgcguguguuuuuguu................    4           0           seq
```
